# Supplementary material for: Vti1a/b support distinct aspects of TGN and cis-/medial Golgi organization
Source: Sci Rep. 2022 Dec 2;12:20870. doi: 10.1038/s41598-022-25331-x (PMC9718741; doi:10.1038/s41598-022-25331-x)
Supplement: Supplementary file 1 — Supplementary Information. [file 41598_2022_25331_MOESM1_ESM.pdf]

Vti1a/b support distinct aspects of TGN and *cis-*/medial Golgi organization

Danique M van Bommel[1], Ruud F Toonen[1] and Matthijs Verhage*[1,2]

Supplementary information

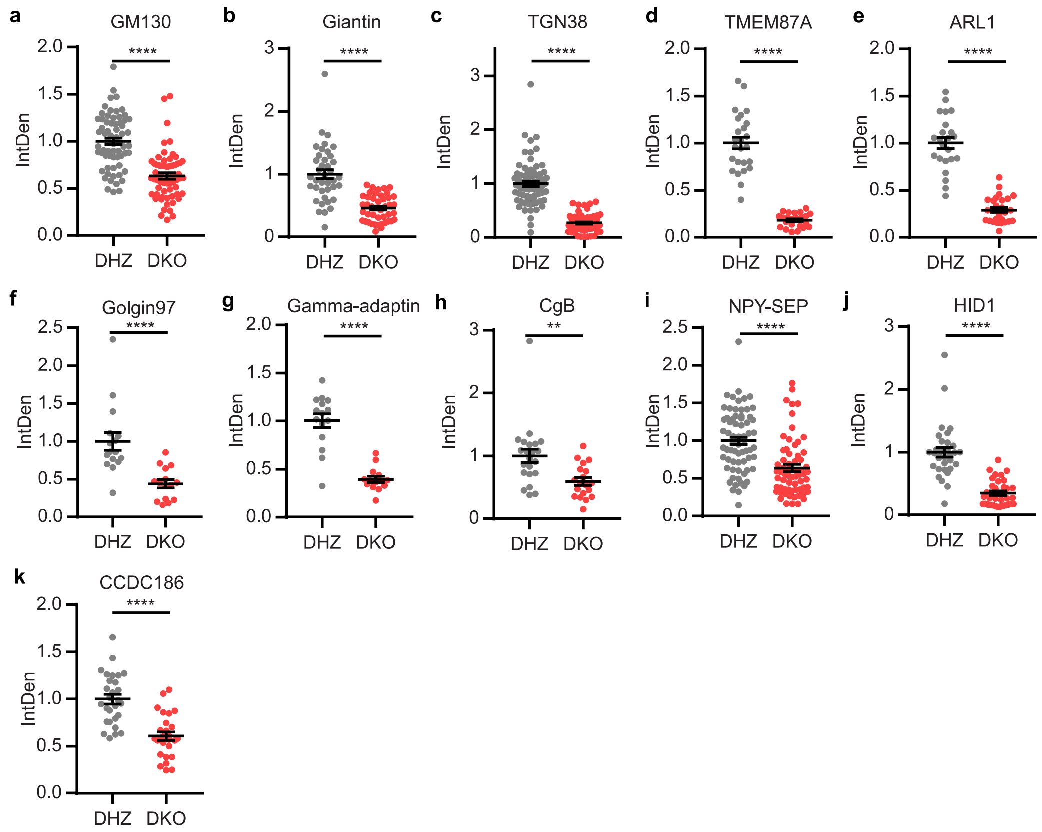

Supplementary Figure 1: Golgi protein levels are lower in Vti1a/b DKO neurons. a-i Integrated density (IntDen; sum of all pixel values) of GM130 (DHZ: n = 65; DKO: n = 61). Mann Whitney test: ****p < 0.0001. (**a**), giantin (DHZ: n = 39; DKO: n = 44). Mann Whitney test: ****p < 0.0001. (**b**), TGN38 (DHZ: n = 73; DKO: n = 69). Mann Whitney test: ****p < 0.0001. (**c**), TMEM87A (DHZ: n = 25; DKO: n = 23). *t*-test: ****p < 0.0001. (**d**), ARL1 (DHZ: n = 23; DKO: n = 27). *t*-test: ****p < 0.0001. (**e**), Golgin97 (DHZ = 16; DKO = 15). Mann Whitney test: ****p < 0.0001 (**f**), gamma-adaptin (DHZ = 15; DKO = 14). *t*-test: ****p < 0.0001. (**g**) CgB (DHZ: n = 22; DKO: n = 19). Mann Whitney test: **p = 0.0011. (**h**), NPY-SEP (DHZ: n = 67; DKO: n = 64). Mann Whitney test: ****p < 0.0001. (**i**), HID1 (DHZ: n = 32; DKO: n = 35). Mann Whitney test: ****p < 0.0001. (**j**) and CCDC186 (DHZ: n = 28; DKO: n = 27). *t*-test: ****p < 0.0001. (**k**) in the Golgi of Vti1a/b DKO neurons, normalized to DHZ controls. Bars show mean ± SEM. Detailed statistics are shown in Supplementary Table 1.

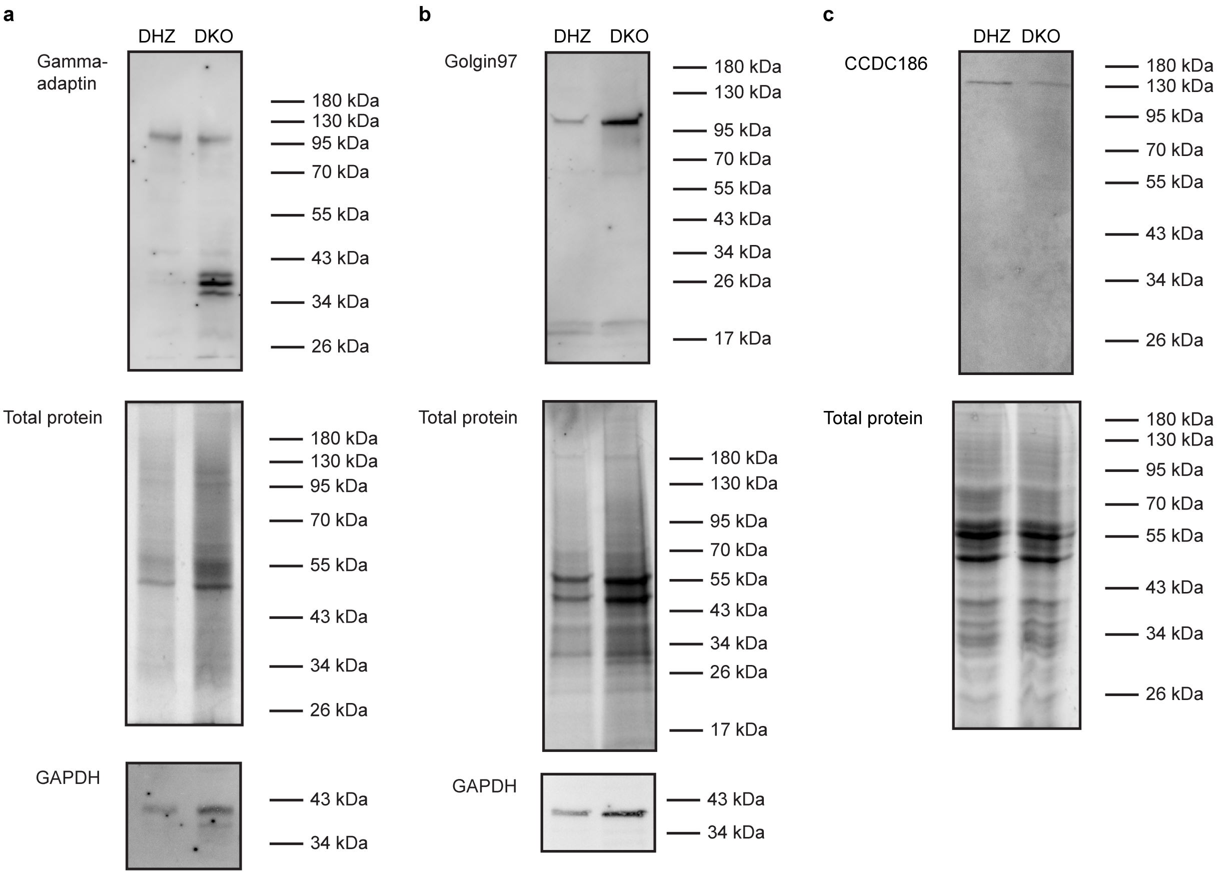

Supplementary Figure 2: Western blot results are in line with immunofluorescence data. a Western blot for gamma-adaptin, total protein staining and GAPDH. **b** Western blot for Golgin97, total protein staining and GAPDH. **c** Western blot for CCDC186 and total protein staining. Full size blots and gels are shown in Supplementary Figure 3.

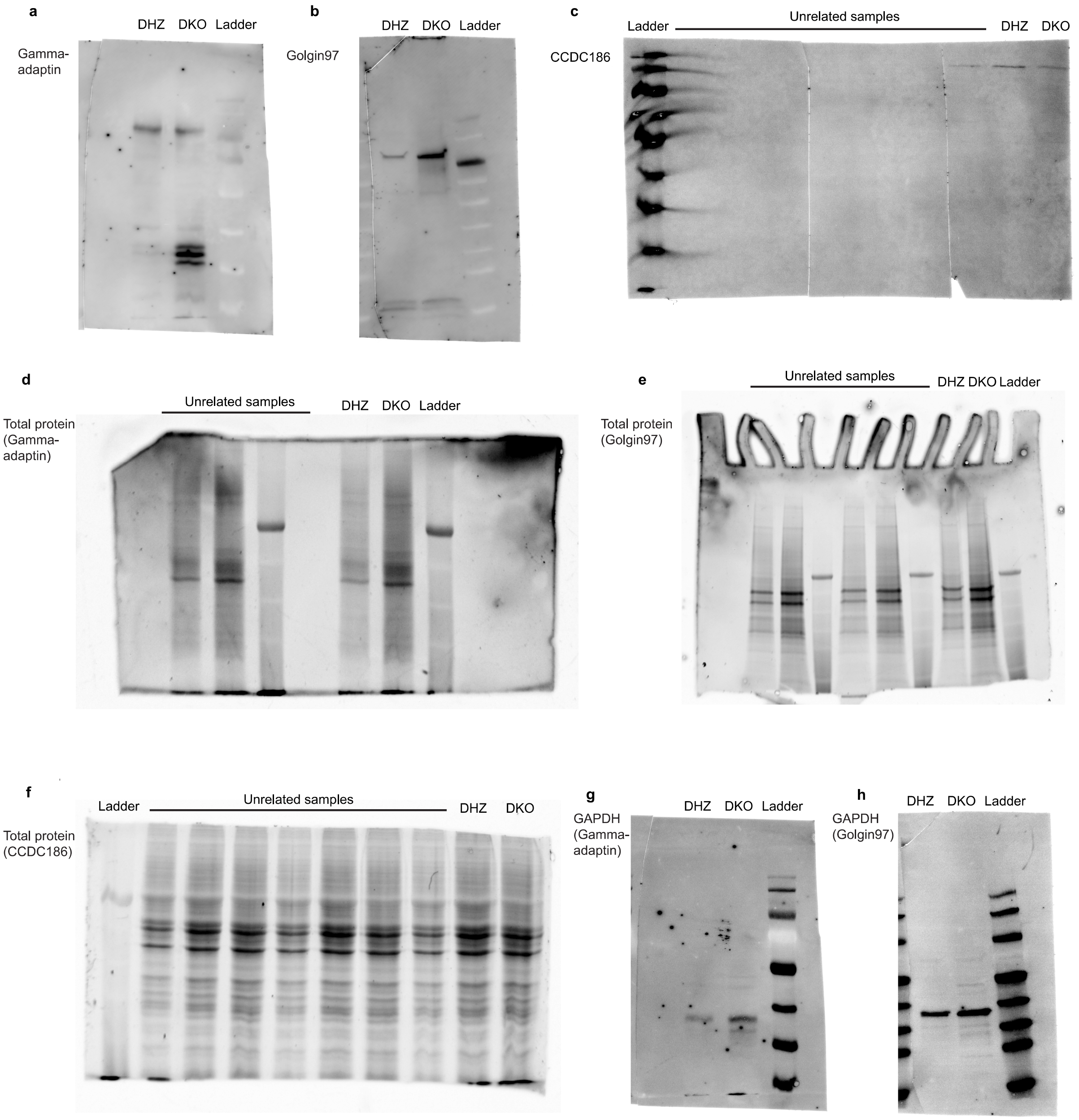

Supplementary Figure 3: Full size images of Western blots and gels in Supplementary Figure 2. a-c Full size Western blots of gamma-adaptin (**a**), Golgin97 (**b**) and CCDC186 (**c**). **d-f** Full size gels showing total protein staining of samples for gamma-adaptin (**d**), Golgin97 (**e**) and CCDC186 (**f**). **g-h** Full size Western blots showing GAPDH staining of samples for gamma-adaptin (**g**) and Golgin97 (**h).**

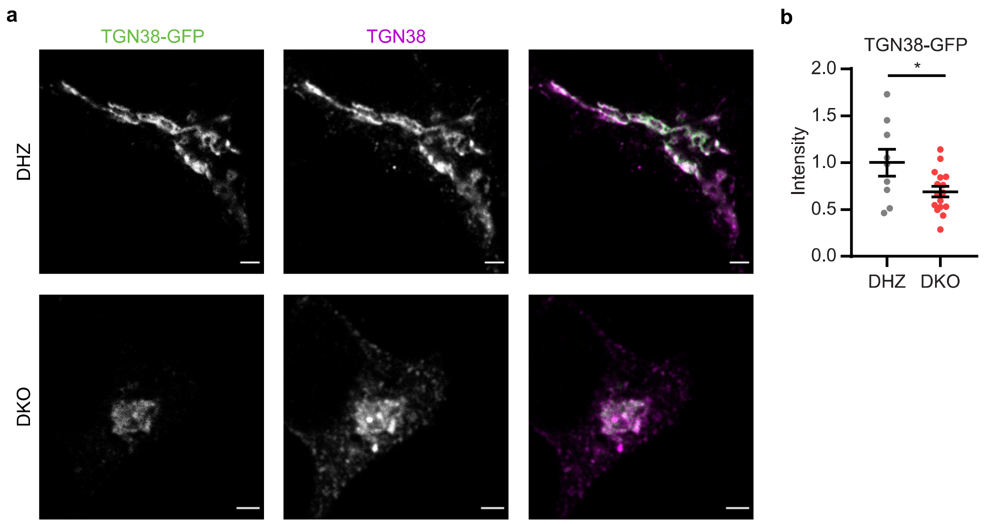

Supplementary Figure 4: Decreased TGN staining intensity in Vti1a/b deficient neurons is not caused by epitope masking. a Representative examples of neurons overexpressing TGN38-GFP, immunostained for TGN38. **b** Normalized staining intensity of TGN38-GFP in the Golgi is decreased (DHZ: n = 9; DKO: n = 16). *t*-test: *p = 0.0268. Bars show mean ± SEM. Detailed statistics are shown in Supplementary Table 1. Scale bar is 2 μm.

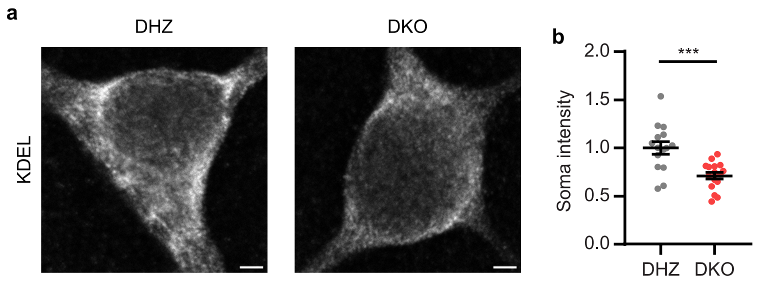

Supplementary Figure 5: Decreased KDEL staining intensity in Vti1a/b DKO neurons. a Representative examples of neurons immunostained for KDEL. **b** KDEL normalized staining intensity in the soma is decreased (DHZ: n = 15; DKO: n = 16). *t*-test: ***p = 0.0004. Bars show mean ± SEM. Detailed statistics are shown in Supplementary Table 1. Scale bar is 2 μm.

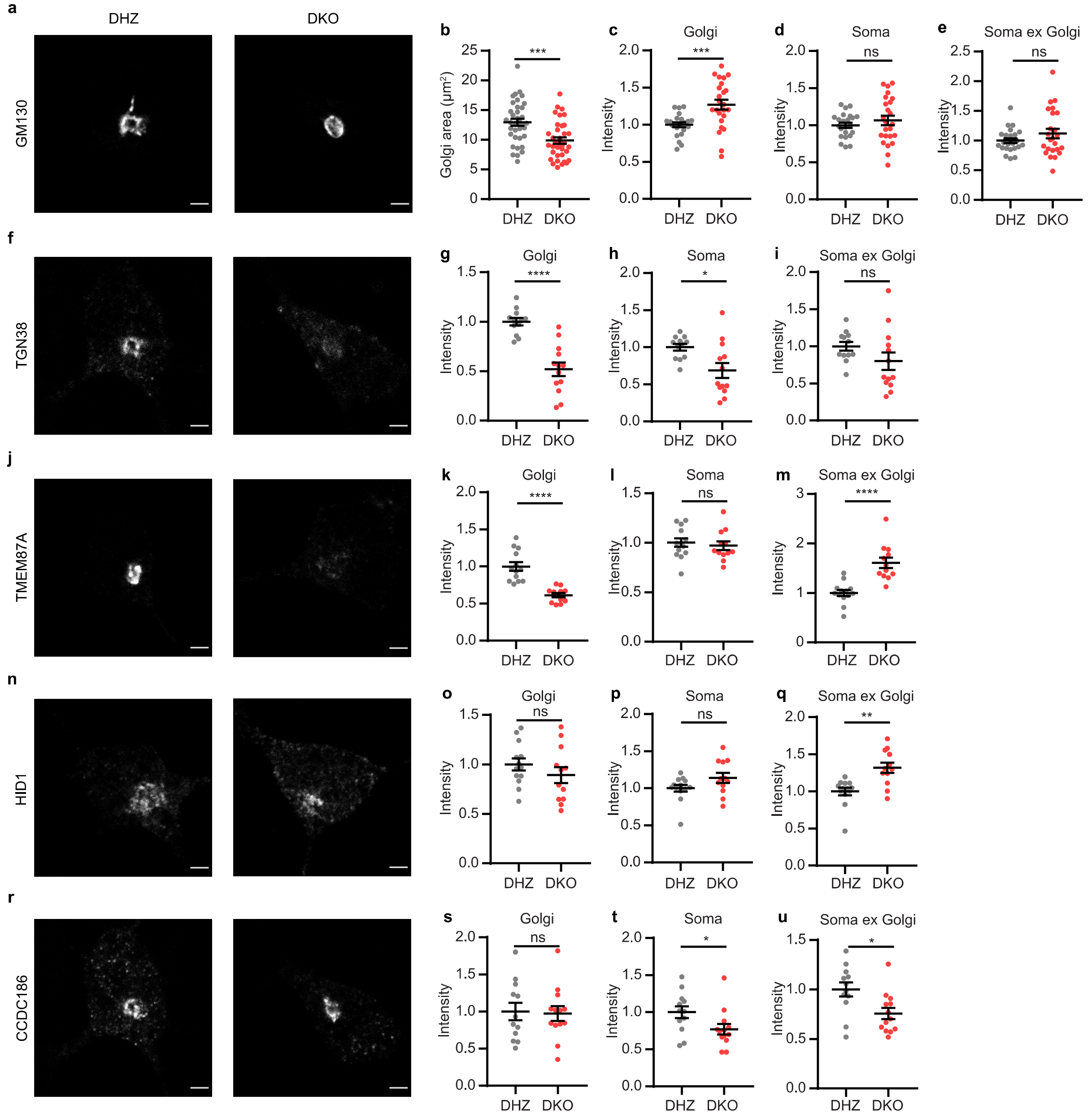

Supplementary Figure 6: DIV2 Vti1a/b deficient neurons show similar phenotype as DIV8 neurons. a Representative examples of DIV2 neurons immunostained for GM130. **b** Decreased Golgi area in DIV2 Vti1a/b DKO neurons, compared to DHZ controls (DHZ: n = 36; DKO: n = 36). *t*-test: ***p = 0.0002. **c-e** GM130 normalized staining intensity is increased in the Golgi (DHZ: n = 23; DKO: n = 24). *t*-test: ***p = 0.0007. (**c**) and not significantly different in the soma (DHZ: n = 23; DKO: n = 24). *t*-test: ns, p = 0.3702. (**d**) and soma excluding Golgi (DHZ: n = 23; DKO: n = 24). *t*-test: ns, p = 0.1899. (**e**). **f** Representative examples of DIV2 neurons immunostained for TGN38. **g-i** TGN38 normalized staining intensity is decreased in the Golgi (DHZ: n = 12; DKO: n = 13). *t*-test: ****p < 0.0001. (**g**) and soma (DHZ: n = 12; DKO: n = 13). *t*-test: *p = 0.0111. (**h**), but not significantly different in the soma excluding Golgi (DHZ: n = 12; DKO: n = 13). *t*-test: ns, p = 0.1513. (**i**). **j** Representative examples of DIV2 neurons immunostained for TMEM87A. **k-m** TMEM87A normalized staining intensity is decreased in the Golgi (DHZ: n = 13; DKO: n = 12). *t*-test: ****p < 0.0001. (**k**), not different in the soma (DHZ: n = 13; DKO: n = 12). *t*-test: ns, p = 0.6244. (**l**) and increased in the soma excluding Golgi (DHZ: n = 13; DKO: n = 12). *t*-test: ****p < 0.0001. (**m**). **n** Representative examples of DIV2 neurons immunostained for HID1. **o-q** HID1 normalized staining intensity is not different in the Golgi (DHZ: n = 13; DKO: n = 12). *t*-test: ns, p = 0.2941. (**o**) and soma (DHZ: n = 13; DKO: n = 12). Mann Whitney test: ns, p = 0.1519. (**p**) and increased in the soma excluding Golgi (DHZ: n = 13; DKO: n = 12). Mann Whitney test: **p = 0.0020. (**q**). **r** Representative examples of DIV2 neurons immunostained for CCDC186. **s-u** CCDC186 normalized staining intensity is not different in the Golgi (DHZ: n = 12; DKO: n = 13). *t*-test: ns, p = 0.8668. (**s**) and decreased in the soma (DHZ: n = 12; DKO: n = 13). *t*-test: *p = 0.0292. (**t**) and soma excluding Golgi (DHZ: n = 12; DKO: n = 13). *t*-test: *p = 0.0136. (**u**).Bars show mean ± SEM. Detailed statistics are shown in Supplementary Table 1. Scale bar is 2 µm.

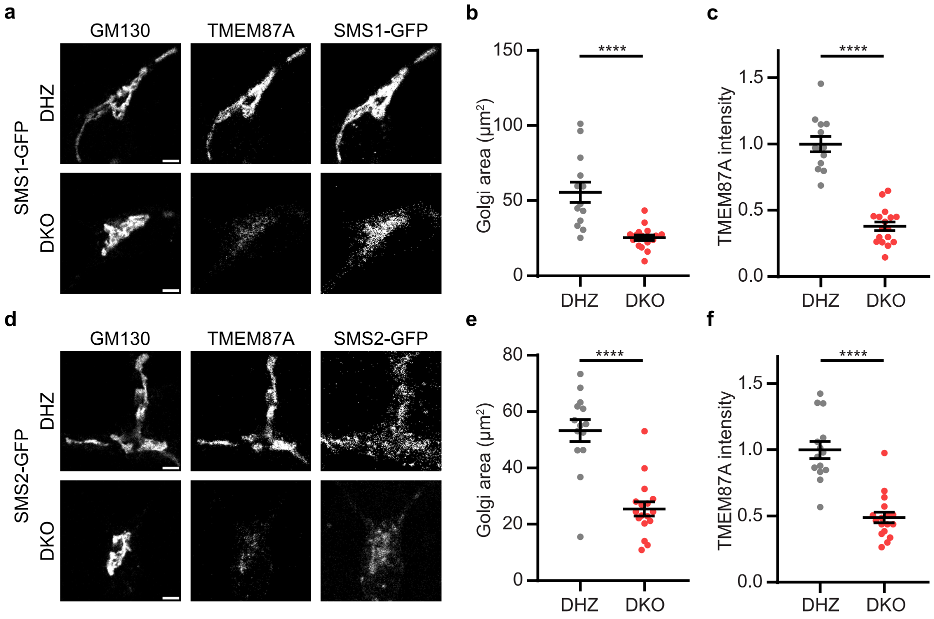

Supplementary Figure 7: Overexpression of SMS1-GFP or SMS2-GFP does not rescue Golgi area or TGN staining intensity in Vti1a/b deficient neurons. a Representative examples of neurons overexpressing SMS1-GFP, immunostained for GM130 and TMEM87A. **b** Golgi area based on GM130 staining remains smaller in Vti1a/b DKO neurons overexpressing SMS1-GFP (DHZ: n = 13; DKO: n = 17). *t*-test: ****p < 0.0001. **c** Normalized staining intensity of TMEM87A in the Golgi remains lower in Vti1a/b DKO neurons overexpressing SMS1-GFP (DHZ: n = 13; DKO: n = 17). *t*-test: ****p < 0.0001. **d** Representative examples of neurons overexpressing SMS2-GFP, immunostained for GM130 and TMEM87A. **e** Golgi area based on GM130 staining remains smaller in Vti1a/b DKO neurons overexpressing SMS2-GFP (DHZ: n = 14; DKO: n = 17). *t*-test: ****p < 0.0001. **f** Normalized staining intensity of TMEM87A in the Golgi remains lower in Vti1a/b DKO neurons overexpressing SMS2-GFP (DHZ: n = 14; DKO: n = 17). Mann Whitney test: ****p < 0.0001. Bars show mean ± SEM. Detailed statistics are shown in Supplementary Table 1. Scale bar is 3 µm.

Supplementary Table 1: Data and statistics. Overview of all data and statistics for each figure. Dataset, condition, average and SEM, the number of independent cells (n), the p-values and statistical tests used are indicated. Statistical tests were two-tailed and used α = 0.05. *p < 0.05; **p < 0.01; ***p < 0.001; ****p < 0.0001. For one-way ANOVA the p-values are also indicated.

Dataset	Condition	Value (Mean ± SEM)	n	p-value	Statistical test
Golgi area GM130 Figure 1b	DHZ	58.7 ± 2.45	64	****p < 0.0001	Mann Whitney test
	DKO	24.4 ± 1.26	66		
GM130 Golgi intensity Figure 1c	DHZ	1 ± 0.0177	65	****p < 0.0001	*t*-test
	DKO	1.49 ± 0.039	61		
Golgi area Giantin Figure 1e	DHZ	54.6 ± 4.59	29	****p < 0.0001	*t*-test
	DKO	21.1 ± 1.25	39		
Giantin Golgi intensity Figure 1f	DHZ	1 ± 0.0445	39	**p = 0.0017	*t*-test
	DKO	1.24 ± 0.0572	44		
TGN38 Golgi intensity Figure 2b	DHZ	1 ± 0.0294	73	****p < 0.0001	Mann Whitney test
	DKO	0.641 ± 0.0476	69		
TGN38 soma intensity Figure 2c	DHZ	1 ± 0.0322	73	****p < 0.0001	Mann Whitney test
	DKO	0.636 ± 0.0416	69		
TGN38 soma ex Golgi intensity Figure 2d	DHZ	1 ± 0.0324	73	*p = 0.0145	Mann Whitney test
	DKO	0.851 ± 0.0581	69		
ARL1 Golgi intensity Figure 2f	DHZ	1 ± 0.0387	23	****p < 0.0001	*t*-test
	DKO	0.756 ± 0.0397	27		
ARL1 soma intensity Figure 2g	DHZ	1 ± 0.0468	23	****p < 0.0001	*t*-test
	DKO	0.653 ± 0.0319	27		
ARL1 soma ex Golgi intensity Figure 2h	DHZ	1 ± 0.0464	23	*p = 0.0212	*t*-test
	DKO	0.851 ± 0.0422	27		
TMEM87A Golgi intensity Figure 2j	DHZ	1 ± 0.0323	25	****p < 0.0001	*t*-test
	DKO	0.462 ± 0.027	23		
TMEM87A soma intensity Figure 2k	DHZ	1 ± 0.0441	25	****p < 0.0001	*t*-test
	DKO	0.461 ± 0.0259	23		
TMEM87A soma ex Golgi intensity Figure 2l	DHZ	1 ± 0.0446	25	ns, p = 0.5162	*t*-test
	DKO	0.955 ± 0.053	23		
Golgin97 Golgi intensity Figure 2n	DHZ	1 ± 0.0655	16	ns, p = 0.4196	*t*-test
	DKO	0.925 ± 0.0646	15		
Golgin97 soma intensity Figure 2o	DHZ	1 ± 0.0604	16	ns, p = 0.0648	*t*-test
	DKO	0.834 ± 0.0622	15		
Golgin97 soma ex Golgi intensity Figure 2p	DHZ	1 ± 0.0604	16	ns, p = 0.1673	*t*-test
	DKO	0.875 ± 0.0648	15		
	DHZ	1 ± 0.0455	15	**p = 0.0067	*t*-test

Gamma-adaptin Golgi intensity Figure 2r	DKO	0.81 ± 0.0459	14		
Gamma-adaptin soma intensity Figure 2s	DHZ	1 ± 0.0566	15	ns, p = 0.0568	Mann Whitney test
	DKO	0.883 ± 0.0445	14		
Gamma-adaptin soma ex Golgi intensity Figure 2t	DHZ	1 ± 0.0614	15	****p < 0.0001	*t*-test
	DKO	1.47 ± 0.0782	14		
CgB Golgi intensity Figure 3b	DHZ	1 ± 0.0724	22	**p = 0.0024	Mann Whitney test
	DKO	1.44 ± 0.132	19		
CgB soma intensity Figure 3c	DHZ	1 ± 0.075	22	ns, p = 0.1142	*t*-test
	DKO	1.16 ± 0.0605	19		
CgB soma ex Golgi intensity Figure 3d	DHZ	1 ± 0.063	22	**p = 0.0056	*t*-test
	DKO	1.25 ± 0.0527	19		
HID1 Golgi intensity Figure 3f	DHZ	1 ± 0.0434	32	****p < 0.0001	*t*-test
	DKO	0.694 ± 0.0398	35		
HID1 soma intensity Figure 3g	DHZ	1 ± 0.0507	32	****p < 0.0001	Mann Whitney test
	DKO	0.62 ± 0.0356	35		
HID1 soma ex Golgi intensity Figure 3h	DHZ	1 ± 0.0532	32	***p = 0.0001	Mann Whitney test
	DKO	0.745 ± 0.0473	35		
CCDC186 Golgi intensity Figure 3j	DHZ	1 ± 0.0267	28	ns, p = 0.2927	*t*-test
	DKO	0.95 ± 0.0394	27		
CCDC186 soma intensity Figure 3k	DHZ	1 ± 0.0275	28	****p < 0.0001	*t*-test
	DKO	0.786 ± 0.0353	27		
CCDC186 soma ex Golgi intensity Figure 3l	DHZ	1 ± 0.0359	28	***p = 0.0006	*t*-test
	DKO	0.805 ± 0.0401	27		
NPY-SEP Golgi intensity Figure 2n	DHZ	1 ± 0.0484	67	ns, p = 0.0567	Mann Whitney test
	DKO	1.2 ± 0.0701	64		
NPY-SEP soma intensity Figure 3o	DHZ	1 ± 0.0509	67	**p = 0.0016	Mann Whitney test
	DKO	0.797 ± 0.0491	64		
NPY-SEP soma ex Golgi intensity Figure 3p	DHZ	1 ± 0.0568	67	*p = 0.0111	Mann Whitney test
	DKO	0.828 ± 0.0527	64		
LAMP1 intensity Figure 4b	DHZ	1 ± 0.0397	16	****p < 0.0001	Mann Whitney test
	DKO	0.606 ± 0.0326	18		
Large oval LAMP1 shapes Figure 4c	DHZ	4.5 ± 0.418	16	****p < 0.0001	Mann Whitney test
	DKO	0.333 ± 0.162	18		
Total oval LAMP1 shapes Figure 4d	DHZ	5.31 ± 0.617	16	****p < 0.0001	Mann Whitney test
	DKO	0.833 ± 0.246	18		

Distance between peaks Figure 5d	(1) DHZ GM130	0.302 ± 0.0184	15	****p < 0.0001: 1 versus 3, 1 versus 4, 2 versus 3, 2 versus 4<br><br>ns, p = 0.3769: 1 versus 2, ns, p = 0.9976: 3 versus 4	One-way ANOVA ****p < 0.0001 (with Tukey's multiple comparisons test)
	(2) DKO GM130	0.346 ± 0.0291	15		
	(3) DHZ TMEM87A	0.0863 ± 0.0112	15		
	(4) DKO TMEM87A	0.09144 ± 0.013	15		
GM130 intensity Figure 6b	(1) DMSO	1 ± 0.0409	22	ns, p = 0.8834	One-way ANOVA (ns, p = 0.8834)
	(2) Cer	0.976 ± 0.0572	16		
	(3) SP	1.02 ± 0.0775	10		
TMEM87A intensity Figure 6d	(1) DMSO	1 ± 0.0702	14	***p = 0.0002: 1 versus 2, ****p < 0.0001: 1 versus 3, ns, p = 0.1312: 2 versus 3	One-way ANOVA (****p < 0.0001) with Tukey's multiple comparisons test
	(2) Cer	0.612 ± 0.0554	10		
	(3) SP	0.43 ± 0.0374	10		
TGN38 intensity Figure 6f	(1) DMSO	1 ± 0.0906	13	*p = 0.0186: 1 versus 2, ***p = 0.0002: 1 versus 3, ns, p = 0.4809: 2 versus 3	One-way ANOVA (***p = 0.0003) with Tukey's multiple comparisons test
	(2) Cer	0.615 ± 0.12	7		
	(3) SP	0.453 ± 0.0522	10		
Golgi area Figure 7b	DMSO	22.7 ± 1.48	44	ns, p = 0.1593	Mann Whitney test
	MYR	26.4 ± 2.05	37		
TMEM87A intensity Figure 7c	DMSO	1 ± 0.0503	35	ns, p = 0.8546	Mann Whitney test
	MYR	0.986 ± 0.0527	27		
Integrated density GM130 Figure S1a	DHZ	1 ± 0.0356	65	****p < 0.0001	Mann Whitney test
	DKO	0.631 ± 0.0331	61		
Integrated density Giantin Figure S1b	DHZ	1 ± 0.0719	39	****p < 0.0001	Mann Whitney test
	DKO	0.459 ± 0.0314	44		
Integrated density TGN38 Figure S1c	DHZ	1 ± 0.0482	73	****p < 0.0001	Mann Whitney test
	DKO	0.267 ± 0.0187	69		
Integrated density TMEM87A Figure S1d	DHZ	1 ± 0.0628	25	****p < 0.0001	*t*-test
	DKO	0.181 ± 0.0153	23		
Integrated density ARL1 Figure S1e	DHZ	1 ± 0.0609	23	****p < 0.0001	*t*-test
	DKO	0.291 ± 0.0265	27		
Integrated density Golgin97 Figure S1f	DHZ	1 ± 0.117	16	****p < 0.0001	Mann Whitney test
	DKO	0.44 ± 0.0549	15		
Integrated density gamma-adaptin Figure S1g	DHZ	1 ± 0.0731	15	****p < 0.0001	*t*-test
	DKO	0.391 ± 0.0327	14		
Integrated density CgB Figure S1h	DHZ	1 ± 0.107	22	**p = 0.0011	Mann Whitney test
	DKO	0.592 ± 0.0612	19		

Integrated density NPY-SEP Figure S1i	DHZ	1 ± 0.0502	67	****p < 0.0001	Mann Whitney test
	DKO	0.64 ± 0.048	64		
Integrated density HID1 Figure S1j	DHZ	1 ± 0.0763	32	****p < 0.0001	Mann Whitney test
	DKO	0.347 ± 0.0343	35		
Integrated density CCDC186 Figure S1k	DHZ	1 ± 0.0511	28	****p < 0.0001	*t*-test
	DKO	0.607 ± 0.0444	27		
TGN38-GFP intensity Figure S4	DHZ	1 ± 0.143	9	*p = 0.0268	*t*-test
	DKO	0.691 ± 0.057	16		
KDEL soma intensity Figure S5b	DHZ	1 ± 0.0633	15	***p = 0.0004	*t*-test
	DKO	0.711 ± 0.0359	16		
Golgi area GM130 Figure S6b	DHZ	13 ± 0.596	36	***p = 0.0002	*t*-test
	DKO	9.88 ± 0.526	36		
GM130 Golgi intensity Figure S6c	DHZ	1 ± 0.0313	23	***p = 0.0007	*t*-test
	DKO	1.27 ± 0.066	24		
GM130 soma intensity Figure S6d	DHZ	1 ± 0.0347	23	ns, p = 0.3702	*t*-test
	DKO	1.07 ± 0.0638	24		
GM130 soma ex Golgi intensity Figure S6e	DHZ	1 ± 0.0416	23	ns, p = 0.1899	*t*-test
	DKO	1.12 ± 0.0805	24		
TGN38 Golgi intensity Figure S6g	DHZ	1 ± 0.0374	12	****p < 0.0001	*t*-test
	DKO	0.52 ± 0.0687	13		
TGN38 soma intensity Figure S6h	DHZ	1 ± 0.0447	12	*p = 0.0111	*t*-test
	DKO	0.689 ± 0.0999	13		
TGN38 soma ex Golgi intensity Figure S6i	DHZ	1 ± 0.0591	12	ns, p = 0.1513	*t*-test
	DKO	0.801 ± 0.116	13		
TMEM87A Golgi intensity Figure S6k	DHZ	1 ± 0.0577	13	****p < 0.0001	*t*-test
	DKO	0.613 ± 0.0283	12		
TMEM87A soma intensity Figure S6l	DHZ	1 ± 0.0439	13	ns, p = 0.6244	*t*-test
	DKO	0.969 ± 0.0445	12		
TMEM87A soma ex Golgi intensity Figure S6m	DHZ	1 ± 0.0618	13	****p < 0.0001	*t*-test
	DKO	1.61 ± 0.106	12		
HID1 Golgi intensity Figure S6o	DHZ	1 ± 0.0606	13	ns, p = 0.2941	*t*-test
	DKO	0.892 ± 0.081	12		
HID1 soma intensity Figure S6p	DHZ	1 ± 0.0472	13	ns, p =0.1519	Mann Whitney test
	DKO	1.14 ± 0.0672	12		
HID1 soma ex Golgi intensity Figure S6q	DHZ	1 ± 0.0511	13	**p = 0.0020	Mann Whitney test
	DKO	1.32 ± 0.0696	12		

CCDC186 Golgi intensity Figure S6s	DHZ	1 ± 0.117	12	ns, p = 0.8668	*t*-test
	DKO	0.974 ± 0.0994	13		
CCDC186 soma intensity Figure S6t	DHZ	1 ± 0.0806	12	*p = 0.0292	Mann Whitney test
	DKO	0.772 ± 0.0713	13		
CCDC186 soma ex Golgi intensity Figure S6u	DHZ	1 ± 0.0718	12	*p = 0.0136	*t*-test
	DKO	0.758 ± 0.0567	13		
SMS1-GFP overexpression Golgi area Figure S7b	DHZ	55.8 ± 6.78	13	****p < 0.0001	*t*-test
	DKO	25.6 ± 1.81	17		
SMS1-GFP overexpression TMEM87A Figure S7c	DHZ	1 ± 0.0567	13	****p < 0.0001	*t*-test
	DKO	0.38 ± 0.0329	17		
SMS2-GFP overexpression Golgi area Figure S7e	DHZ	53.3 ± 3.85	14	****p < 0.0001	*t*-test
	DKO	25.5 ± 2.45	17		
SMS2-GFP overexpression TMEM87A Figure S7f	DHZ	1 ± 0.0649	14	****p < 0.0001	Mann Whitney test
	DKO	0.49 ± 0.0408	17		
